# Habitat-dependent changes in vigilance behaviour of Red-crowned Crane influenced by wildlife tourism

**DOI:** 10.1038/s41598-017-16907-z

**Published:** 2017-11-30

**Authors:** Donglai Li, Yu Liu, Xinghai Sun, Huw Lloyd, Shuyu Zhu, Shuyan Zhang, Dongmei Wan, Zhengwang Zhang

**Affiliations:** 1 Provincal Key Laboratory of Animal Resource and Epidemic Disease Prevention, College of Life Sciences, Liaoning University, Shenyang, 110036 P.R. China; 2 Ministry of Education Key Laboratory for Biodiversity Science and Ecological Engineering, College of Life Sciences, Beijing Normal University, Beijing, 100875 P.R. China; 3 Division of Biology and Conservation Ecology, School of Science and Environment, Manchester Metropolitan University, Chester Street, Manchester, M1 5GD United Kingdom; 4 Yellow River Delta National Nature Reserve Management Bureau, Dongying City, Shandong 257200 China

## Abstract

The Endangered Red-crowned Crane (*Grus japonensis*) is one of the most culturally iconic and sought-after species by wildlife tourists. Here we investigate how the presence of tourists influence the vigilance behaviour of cranes foraging in *Suaeda salsa* salt marshes and *S. salsa/Phragmites australis* mosaic habitat in the Yellow River Delta, China. We found that both the frequency and duration of crane vigilance significantly increased in the presence of wildlife tourists. Increased frequency in crane vigilance only occurred in the much taller *S. salsa/P. australis* mosaic vegetation whereas the duration of vigilance showed no significant difference between the two habitats. Crane vigilance declined with increasing distance from wildlife tourists in the two habitats, with a minimum distance of disturbance triggering a high degree of vigilance by cranes identified at 300 m. The presence of wildlife tourists may represent a form of disturbance to foraging cranes but is habitat dependent. Taller *P. australis* vegetation serves primarily as a visual obstruction for cranes, causing them to increase the frequency of vigilance behaviour. Our findings have important implications for the conservation of the migratory red-crowned crane population that winters in the Yellow River Delta and can help inform visitor management.

## Introduction

As public awareness about the plight of nature increases and digital photography and transportation become more affordable and accessible, recreational activities that involve wildlife watching and photography are becoming more popular worldwide^1,2^. In many developing countries such as China, more and more people are pursuing outdoor wildlife photography as a result of the country’s recent improved economic circumstances^3–5^. Activities such as wildlife photography are widely considered as a means to increase the public awareness of wildlife conservation and natural history by revealing images and videos of animals and their behaviour^3,6^. However, there are concerns that increasing human presence and activity may incur negative effects on wildlife and their native habitats^1,7–10^. Wildlife tourism (e.g. watching and photographing wildlife) serves as a non-consumptive leisure activity concerned with the direct enjoyment of specific species^9,11^, and differs from other forms of human disturbances (e.g. hunting, farming, fishing, walking) in terms of their nature, frequency, and intensity^1,12,13^. Some of the concerns revolve primarily around the numbers of people (e.g. large assemblage of people), the equipment used (e.g. camera shutter noise) and the use/access of transport vehicles (e.g. vehicle movements) coupled with length of time spent under observation. Additionally, many wildlife tourists often directly approach and remain at closer distances to the target species for an undetermined length of time in order to get better view to the animals. Some researchers report that animals may perceive these activities as a form of predation-risk and subsequently alter their physiology (e.g. hormonal stress response, immune system, energetic costs) and behaviour, which may have detrimental consequences^14,15^.

Vigilance is generally defined as the cessation of foraging by an individual to raise its head scanning the immediate environment to monitor for potential threats from predators^16,17^ or conspecifics^18^. Although increasing ‘head-up’ vigilance behaviour will enhance the speed of predator detection, it may also reduce foraging efficiency. This is particular important for species, such as migratory waterbirds which constantly have to maintain their intake rates to meet their energy budget during an extremely limited temporal window of foraging opportunities^19–21^. Some theoretical studies suggest that animals can optimize their trade-offs with their investment in anti-predator vigilance behaviour dictated by short-term changes in predation risk and/or predator types^22,23^. Thus the potential disturbances of wildlife tourism may not resemble a true form of predation-risk to foraging animals and it remains unclear if or to what extent wild individuals will habituate to wildlife watching activities or adapt to this ‘new’ threat with a specialized vigilance strategy.

Vigilance behaviour can also be affected by the characteristics of the immediate habitat^24^ with a number of studies having demonstrated that tall vegetation can serve as a good shelter or refuge by improving crypsis and decreasing vulnerability to predators for foraging animals, causing them to lower their vigilance effort^25–28^. Conversely, tall or dense habitat structures near foraging sites can also increase visual obstruction for foraging animals, thus increasing predation risk and vigilance^29–35^. Some bird species are known to increase their vigilance level by 13% when foraging in visually-obstructed vegetation patches compared to more open areas that permit a clearer field of view^36^. Studies have concluded that the regular use of a ‘head-up’ behaviour is indicative that the animals view within or around the foraging habitat is particularly important for wild animals to assess the potential risk^36,37^. Fewer studies have examined the anti-predator vigilance ability of an animal’s ‘head-down’ posture whilst foraging or determined whether foraging animals can reduce their head-up vigilance by increasing the level of detection during head-down foraging in more open habitats. Many spectacular assemblages of birds are now the focal point of many nature photography and tourism, and some of the most sought after species include rare or endangered migratory waterbirds^3^. Flocks of Red-crowned Cranes (*Grus japonensis*) regularly attract hundreds or thousands of photographers annually, particularly at a number of accessible stop-over sites within their range during the migratory season. Red-crowned Crane is globally ‘Endangered’ (IUCN 2016), having undergone a serious population decline in China^38^. The Yellow River Delta, which is located on the south of Bohai Bay, is one of the most important stopover sites for red-crowned cranes, where the cranes prefer to forage in short *Suaeda salsa* vegetation to seek out nutrient-rich tidal mudflat crabs^19^. However, in the past decade, tidal *S. salsa* vegetation has undergone extensive succession to *Phragmites australis* - *Suaeda salsa* habitat, which represents a sub-optimal foraging habitat for the migratory cranes with a significantly lower crab prey resource base (Li *et al*. unpublished data). Whether cranes are exposed to some form of disturbance by nature photographers/wildlife watchers in this wetland habitat mosaic, whether the taller *P. australis* vegetation provide the cranes with sufficient shelter or act as a visual obstruction, or how these vegetation changes may influence crane vigilance behaviour remains unknown.

**Figure 1:**
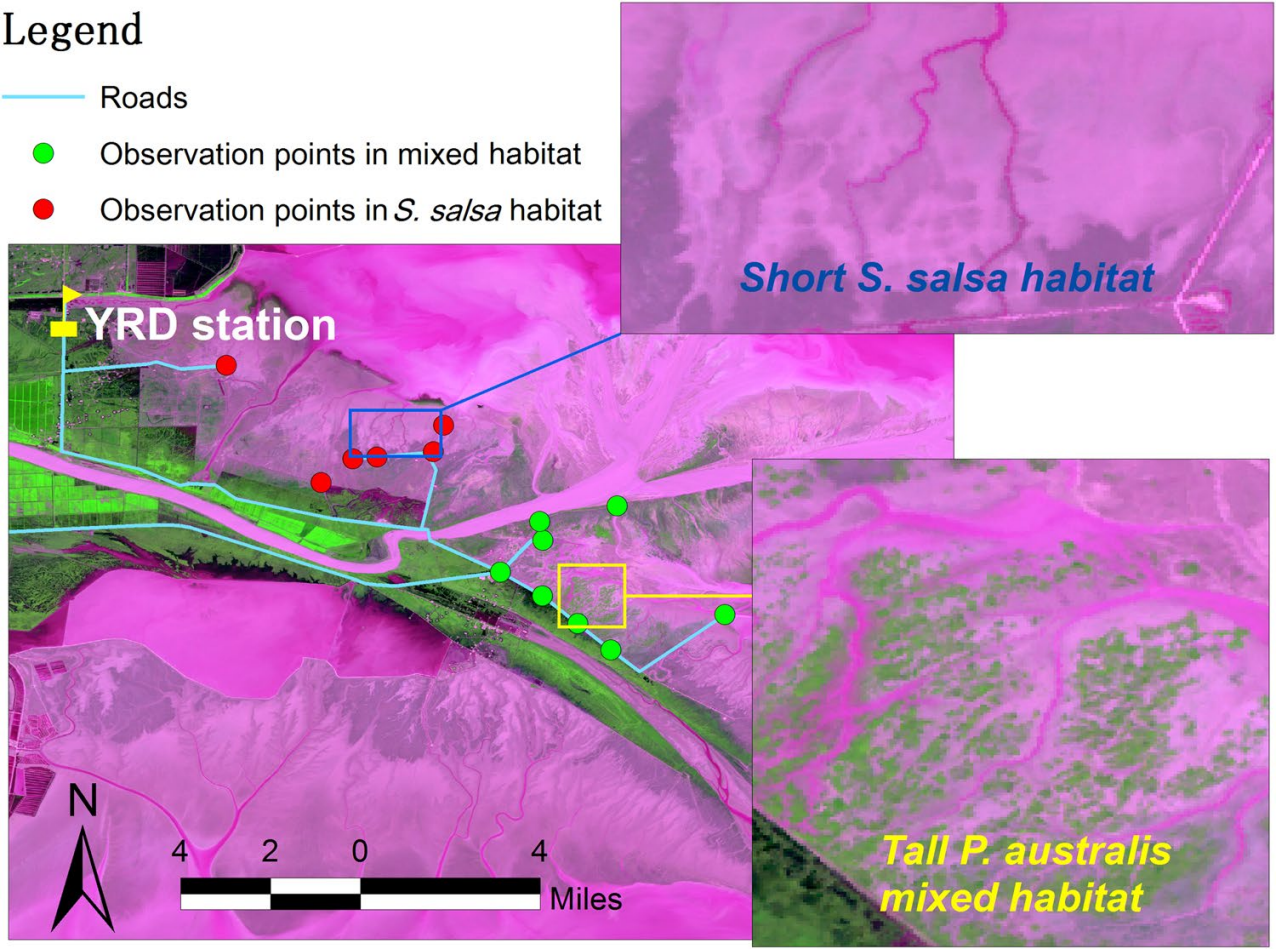
Location of the two wetland habitats within the Yellow River Delta. The green patch shown in the yellow square represents *P. australis* vegetation which is interspersed with areas of shorter *S. salsa* (dark grey). The dark grey in the blue square is the vegetation of *S. salsa*, part of which is submerged by tidal waters (purple colour). The satellite image was obtained on July 8, 2008 (Landsat TM).

In this study, we explore how human activity in the form of wildlife tourism (i.e. bird photography and bird watching) affects the vigilance behaviour of migratory Red-crowned Cranes in two different foraging habitats - *S. salsa* salt marshes (hereafter short *S. salsa* habitat) and a mosaic of *S. salsa* - *P. australis* habitat (hereafter taller P. australis mosaic habitat) (Fig. 1). We hypothesized that: (i) foraging cranes would increase their vigilance in the presence of wildlife tourist activitites; (ii) the impact of wildlife tourist presence on crane vigilance differs between the two foraging habitats i.e. the presence of wildlife tourists would increase vigilance if *P. australis* vegetation acts as a visual obstruction, or reduce vigilance by cranes if the *P. australis* functions as a refuge. In addition, flock size, age structure and the distance from the observer or nearest road to the individuals under observations were also considered when assessing the changes in crane vigilance behaviour to wildlife tourist presence.

## Results

In total, we successfully obtained 244 focal observations (lasting 2,158 minutes) of crane behaviour satisfying for the data analysis (i.e., seeing some cranes easily, not disturbed by other kinds of human activities during observations). One hundred and fifty-four observations (63.1%) were obtained from the shorter coastal *S. salsa* salt marsh habitat while 90 observations (36.9%) were obtained from the tall *P. australis* mosaic habitat (Table 1). Mean observation time per focal individual for cranes in the short *S. salsa* vegetation habitat (8.53 ± 3.33 min) was significantly shorter than observation time of cranes from the taller *P. australis* mixed habitat (9.39 ± 2.87 min; t = 2.045, df = 242, *P* = 0.042). Mean percentage time of cranes were obstructed by habitat features in the taller *P. australis* mosaic (15.46 ± 24.65%, n = 80) was significant longer than that in the shorter *S. salsa* habitat (3.83 ± 14.53%, n = 123; Mann-Whitney U test: z = 4.735, *P* < 0.0001). Thirty-one observations of wildlife tourist presence were recorded, of which 23 were in the presence of bird photographers while eight were collected in the presence of bird watchers. Mean distance between the wildlife tourist and the focal crane was 358.1 ± 287.5 m, significantly closer than the distance between the nearest road (potential disturbance) and the crane observed in the absence of wildlife tourists (495.9 ± 305.5 m; z = 2.534, *P* = 0.011). However, we found no significant difference in the distance between wildlife tourists and the focal crane between the taller *P. australis* mosaic habitat (410.0 ± 334.2 m) and shorter *S. salsa* habitat (340.0 ± 275.4 m; z = 0.409, *P* = 0.707). The mean value and sample size for each sub-group of other explaining factors see Table 1.

**Table 1:** Mean value ± SD of the vigilance frequency and percentage of time spent on vigilance by individual red-crowned cranes for each sub-group of fixed and other explaining factors.

Fixed and other explaining factors		Vigilance frequency (/min)	Time spent vigilant (%)	Sample size
Habitat	*S. salsa* habitat	1.35 ± 1.05	20.59 ± 20.24	n = 154
	Tall *P. australis* mosaic habitat	1.67 ± 1.56	18.93 ± 17.67	n = 90
Wildlife tourists	Yes	2.07 ± 1.64	30.31 ± 22.71	n = 31
	no	1.38 ± 1.19	18.47 ± 18.34	n = 213
Age	Adult	1.66 ± 1.37	21.95 ± 20.05	n = 182
	Juvenile	0.93 ± 0.68	14.17 ± 15.70	n = 62
Migratory season	Autumn	1.27 ± 0.91	18.34 ± 18.04	n = 86
	Spring	1.58 ± 1.42	20.87 ± 19.96	n = 158
Time of day	Morning	1.55 ± 1.49	21.70 ± 22.06	n = 77
	Noon	1.27 ± 0.88	18.16 ± 16.90	n = 127
	Afternoon	1.94 ± 1.69	22.42 ± 20.71	n = 40
Family size	Family without juvenile	1.83 ± 1.64	22.51 ± 20.81	n = 50
	Family with one juvenile	1.46 ± 1.04	20.35 ± 18.23	n = 83
	Family with two juveniles	1.37 ± 1.27	19.86 ± 20.17	n = 96
	Uncertain family in the flock	0.98 ± 0.71	22.42 ± 20.71	n = 15

**Figure 2:**
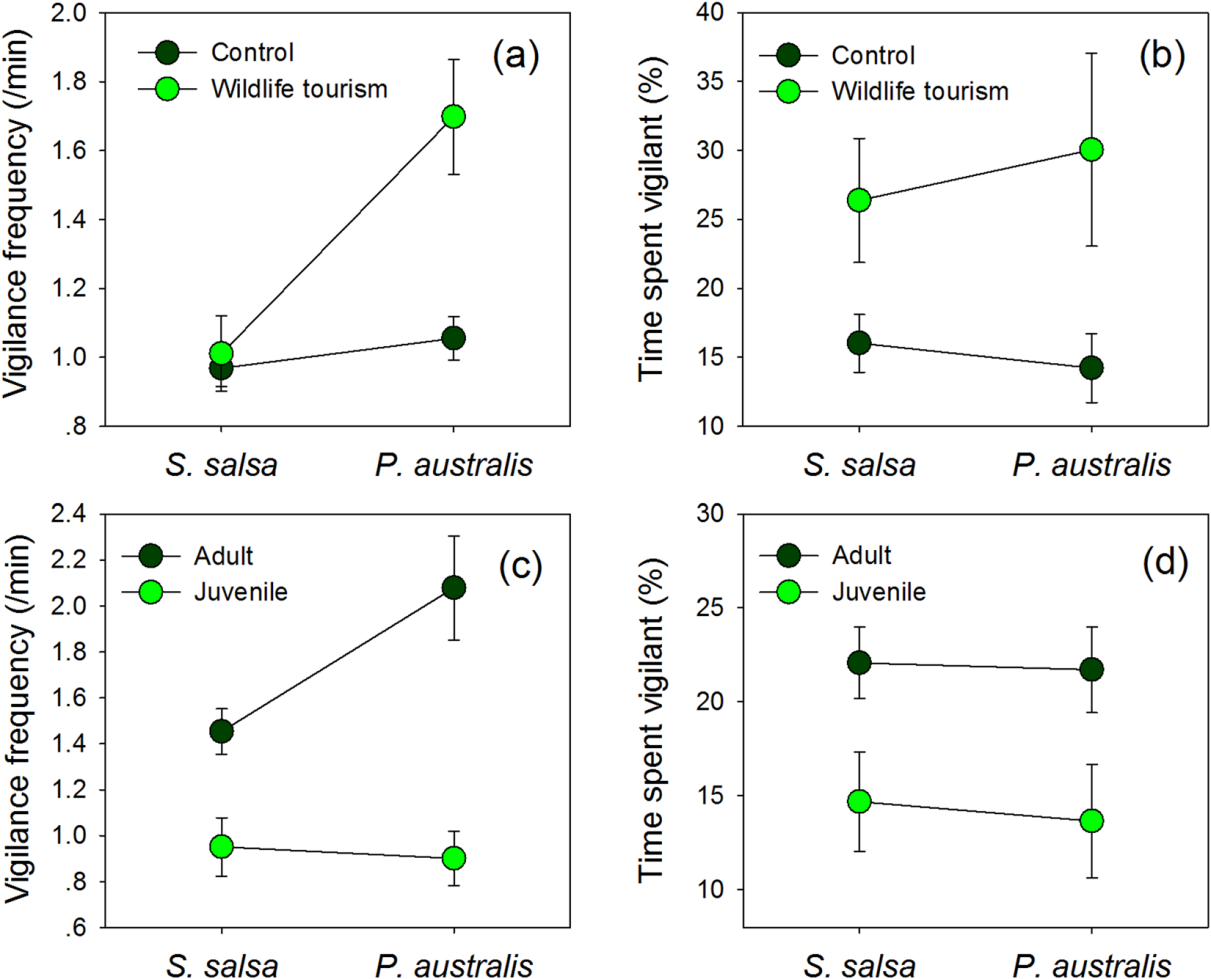
Effects of presence of wildlife tourists and age-class on the vigilance behaviour of red-crowned cranes foraging in the shorter *S. salsa* habitat and taller *P. australis* mosaic habitat. Illustrated values are the least-squares means and standard errors of vigilance frequency and vigilance time.

**Figure 3:**
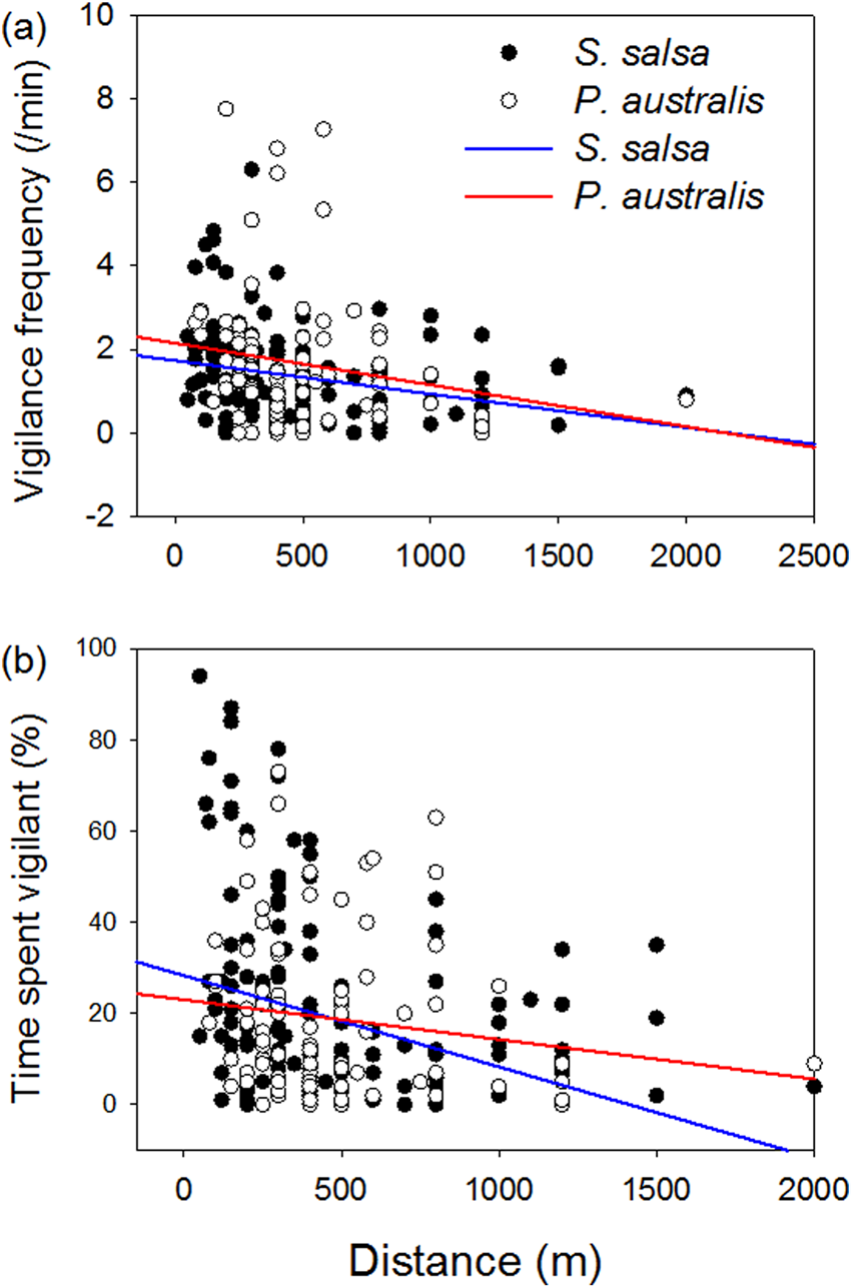
Effect of the distance to the nearest road on crane vigilance (**a**): frequency of vigilance behaviour; (**b**): percentage of time spending on vigilance behaviour of red-crowned cranes in shorter *S. salsa* (filled circles), and tall *P. australis* mosaic wetland habitat (open circles), Yellow River Delta. The blue and red lines represent the linear regression curves between the vigilance and the distance in the shorter *S. salsa* habitat and tall *P. australis* mosaic respectively.

**Figure 4:**
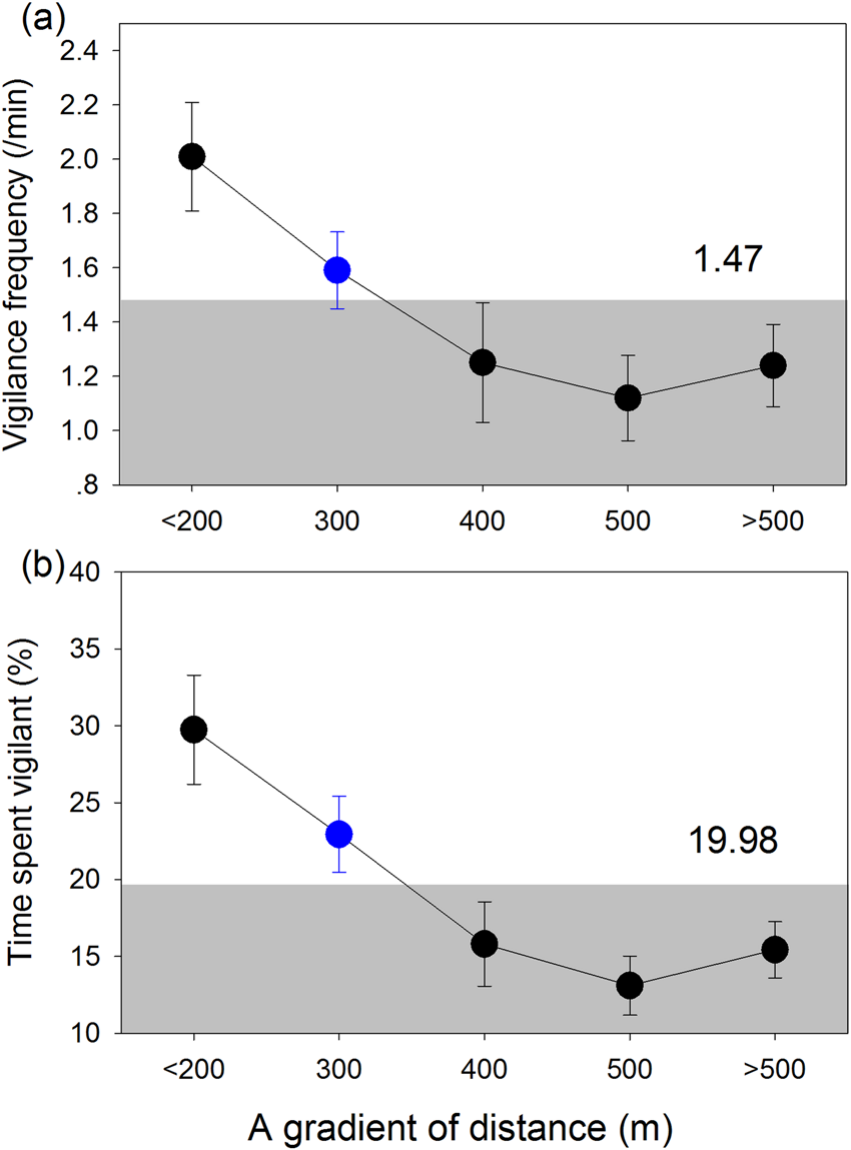
Frequency of vigilance and percentage of time spent on vigilance behaviour by individual red-crowned cranes at different distance intervals from wildlife tourists. Circule with blue denotes significant difference between two adjoining gradient of distance (post-hoc Student-Neuman-Keuls (S-N-K) test, P < 0.05). Block diagram with gray denotes samples below the average level of vigilance (vigilance frequency: 1.47/min; time spent vigilant: 19.98%). Value was showed as Mean ± SE.

We used the method of backward deletion of non-significant contributed terms to build the GLMM model (See methods). The final model for the vigilance frequency revealed a significant effect of habitat (F_1,238_ = 14.479, *P* < 0.001), presence of wildlife tourists (F_1,238_ = 10.937, *P* < 0.001), distance to roads (F_1,238_ = 12.684, *P* < 0.001), age-class (adult or juvenile) (F_1,238_ = 23.777, *P* < 0.001), and the interaction between the habitat and presence of wildlife tourists (F_1,238_ = 8.728, *P* = 0.003). The number of head-up vigilances per min by individual cranes was higher in the presence of wildlife tourists [back-transformed means ± sd (95% C.I.): 1.83 ± 0.87 (1.32, 2.43)] than in the absence of wildlife tourists [1.02 ± 0.56 (0.84, 1.22)] and the difference was much larger in the taller *P. australis* mosaic when wildlife tourists were present [2.88 ± 0.91 (1.88, 4.10)] than when tourists were absent [1.11 ± 0.60 (0.86, 1.39); Fig. 2a; Table 1]. The estimated number of vigilance behaviours per min in the taller mosaic habitat [1.89 ± 0.81(1.43, 2.42)] was also higher than that in the shorter *S. salsa* habitat [0.98 ± 0.52 (0.74, 1.25)], and higher for adult cranes [1.80 ± 0.63 (1.50, 2.13)] than juveniles [1.05 ± 0.41 (0.76, 1.39)], which showed no significant difference between the two habitat types (F_1,230_ = 1.541, *P* = 0.216; Fig. 2c). Frequency of vigilance behaviour decreased by 0.175 ± 0.99 (95% C.I.: 0.078–0.272) with increasing distance (log transformed) between the focal crane and the nearest road (Table 2), whilst the decrease showed no significant differences between the two habitats (F_1,214_ = 0.030, *P* = 0.862; Fig. 3a). As a result, if these two factors were considered together, the vigilance frequency of cranes were significant higher when wildlife tourists were present within 300 m of individual cranes (≥1.50 ± 1.07) than beyond 300 m (≤1.25 ± 1.43; Fig. 4a). Other potential factors had no significant effect on vigilance frequency (season: F_2,216_ = 0.244, *P* = 0.784; time of day: F_2,233_ = 2.684, *P* = 0.070; and family group: F_3,218_ = 0.698, *P* = 0.554). Consequently these factors were not included in the final model.

**Table 2:** Effects of habitat type, presence of wildlife tourists, age class and the distance to the nearest road on the vigilance behaviour of red-crowned cranes in the Yellow River Delta.

Dependent variable	Term	Estimate	SE	T	p	95% confidence interval	
						low	high
Frequency of vigilance (/min)	**Intercept**	**2.579**	**0.322**	**8.015**	**<0.001**	**1.945**	**3.212**
	**habitat** ^**a**^						
	***Suaeda salsa***	**−0.687**	**0.190**	**−3.609**	**<0.001**	**−1.062**	**−0.312**
	**Disturbance** ^**b**^						
	**No**	**−0.643**	**0.175**	**−3.677**	**<0.001**	**−0.988**	**−0.299**
	**ln(distance)**	**−0.175**	**0.049**	**−3.561**	**<0.001**	**−0.272**	**−0.078**
	**Age** ^**c**^						
	**Adult**	**0.318**	**0.065**	**4.876**	**<0.001**	**0.189**	**0.446**
	habitat*disturbance	**0.601**	**0.203**	**2.954**	**0.003**	**0.200**	**1.001**
Time spent vigilant (%)	**Intercept**	**0.968**	**0.135**	**7.155**	**<0.001**	**0.701**	**1.234**
	habitat^a^						
	*Suaeda salsa*	**−**0.113	0.080	**−**1.403	0.162	**−**0.271	0.046
	**Disturbance** ^**b**^						
	**No**	**−0.185**	**0.073**	**−2.522**	**0.012**	**−0.329**	**−0.04**
	**ln(distance)**	**−0.081**	**0.021**	**−3.874**	**<0.001**	**−0.121**	**−0.04**
	**Age** ^**c**^						
	**Adult**	**0.099**	**0.027**	**3.740**	**<0.001**	**0.047**	**0.152**

notes: ^a^the reference category for habitat is –the taller *P. australis* mosaic habitat; ^b^the reference category for disturbance is the presence of human disturbance; ^c^the reference category forage is juvenile. Test statistics and P-values for non-significant terms are from the backward elimination procedure just before the particular term (being the least significantly correlated) was removed from the model. Results of significant predictors from the final models are highlighted in bold.

Foraging cranes spent approximately 20% of their time exhibiting vigilance behaviour. The final GLMM model for time spent on vigilance revealed significant effects from the presence of wildlife tourists (F_1,238_ = 8.012, *P* = 0.005), the distance to roads (F_1,238_ = 15.004, *P* < 0.001) and age-class of the crane (F_1,238_ = 13.990, *P* < 0.0001), but not habitat type (F_1,238_ = 1.488, *P* = 0.224). There was no significant difference in the percentage of time spent on vigilant behaviour between shorter *S. salsa* salt marsh and the taller *P. australis* mosaic habitat [back-transformed means (95% C.I.): 15.6% (11.7%, 20.0%) vs 20.0% (14.0%, 27.1%), respectively]. The estimated time spent on vigilance by cranes increased in the presence of wildlife tourists relatively to the absence of tourists [12.9% (10.5%, 15.5%) vs 23.3% (15.9%, 32.3%), respectively], even though there was no significant effects from the interaction between habitat and wildlife tourist presence (F_2,238_ = 1.979, *P* = 0.161; Fig. 2b). Estimated time spent on vigilance was also higher for adult cranes than juveniles (22.2% (18.0%, 26.7%) vs 13.8% (9.5%, 18.7%), respectively), and showed similar patterns in the two habitats without significant effect from the interaction between the habitat and age of crane (F_1,218_ = 0.174, *P* = 0.677; Fig. 2d). The estimated vigilance time of cranes decreased by 8.1 ± 4.2% (95% C.I.: 4.0–12.1%) with increasing the distance (log transformed) between the crane foraging site and the nearest road, for both habitats combined (Table 2) and each habitat separately (Fig. 3b). There was also no significant effect from the interaction between habitat and the distance to roads (F_1,216_ = 0.036, *p* = 0.851). The cranes spent more time in vigilance when the wildlife tourists were present within 300 m (≥25.0 ± 18.7%) than beyond 300 m (≤15.8 ± 17.8; Fig. 4b). Other confounding factors had no effects on the time spent on vigilance (season: F_2,226_ = 1.426, *P* = 0.242; time of day: F_2,226_ = 1.477, *P* = 0.231; and family group: F_3,233_ = 2.362, *P* = 0.072) and these were removed during the construction of final model.

## Discussion

Our study showed that the wildlife tourists (bird watchers and nature photographers) constituted a form of disturbance to foraging red-crowned cranes in the Yellow River Delta Nature Reserve. This evidence supports our first hypothesis that wildlife tourism caused cranes to increase their vigilance efforts in a manner similar to that for other kinds of disturbances (e.g. agricultural activities^39,40^; fishing^41^). Several previous studies have shown that the behaviour of wild birds can be affected by many different kinds of human activities^7,39,42^. One previous study found that red-crowned cranes significantly increase their vigilance in farmed wetland landscapes such as rice fields and fish ponds in the Yancheng Nature Reserve, compared to other areas of the reserve where human presence is strictly prohibited^41^. However, two other studies have reported that birdwatchers have a minimal impact on the vigilance behaviour of sandhill cranes (*Grus canadensis*) at their spring stopover sites in America^43,44^. Studies of brown bears (*Ursus arctos*) have shown that bears perceive tourists as a risk and respond with changes in vigilance or displacement^45,46^. Consequently wildlife tourism activities based around crane populations in the YRD should be carefully monitored and the balance between viewing opportunities and the need of wildlife populations to forage undisturbed be carefully considered^47^.

We found that presence of wildlife tourists had different effects on the vigilance of red-crowned cranes in different wetland habitats. Crane vigilance frequency increased in the taller *P. australis* mosaic habitat in the presence of wildlife tourists, while remaining unchanged in the short *S. salsa* habitat. In addition, crane vigilance frequency in the tall *P. australis* mixed habitat was significantly higher than that in the short *S. salsa* habitat. Thus, taller *P. australis* vegetation represented a visual obstruction to the detection of potential threats rather than serve as a protective measure for foraging cranes. Similar findings have been reported in a number of studies on granivorous passerines^36^ and specific species such as semipalmated sandpiper *Calidris pusilla* ^48^. Our study showed the effect on the vigilance behaviour of red-crowned cranes caused by the obstruction of *P. australis* was only apparent when examining vigilance frequency data, and not using time budget analyses. This particular vigilance strategy might be explained by the fact that the red-crowned cranes treat the presence of wildlife tourists at the Yellow River Delta as a low-level threat which requires minimal allocation of time to vigilance, or they might exhibit some habituation to these kinds of human presence. Due to limited sample sizes we were unable to determine whether cranes respond differently to different tourism activities e.g. photography and bird-watching, and this merits further work.

A number of studies have advocated the use of vigilance behaviour during wildlife tourist viewing activities as a non-invasive and simple method to monitor wildlife behaviour since it resembles predator avoidance^49,50^. However, others have suggested the importance of considering the context in which vigilance occurs^51^ e.g. vigilance may serve simply to observe conspecifics who may compete for food resources^52,53^. Thus, other behavioural responses such as taking flight, flight initiation distance and displacement from the foraging site should also be the focus of future studies. In particular, flushing birds, which regularly happens at the YRD may have considerably higher impacts on cranes in terms of energetic costs or lost foraging opportunities than vigilance behaviour alone. Future studies would also benefit from linking observed vigilance levels to some body condition or health index of the cranes to quantify actual impact and further inform local conservation management.

One other reason for the relative low frequency of vigilance behaviours in the shorter *S. salsa* habitat under human disturbance might be due to specific adjustments of the cranes’ vigilance posture in response to the non-obstructive low vegetation. Some studies have suggested that some level of vigilance can be maintained when the head of the bird is not held up, provided there is no visual obstruction^54^. Birds have considerable ability to detect approaching threats even when they are not overtly vigilant, although their detection ability is lower than that with raised heads^37,54^. The reason for the low vigilance frequency displayed by the red-crowned cranes in the shorter *S. salsa* habitat may be due to the cranes being able to observe their immediate environment during their ‘head-down’ foraging posture in the shorter *S. salsa* vegetation, which could still provide a good view without the need to engage in frequent head-up vigilance. However, the situation is quite different in the case of the more obstructive *P. australis* vegetation where cranes would need to increase their head-up vigilance frequency in order to gain enough information about potential threats.

Similar to other studies^55–57^, we found that juvenile red-crowned cranes were in a less frequent state of vigilance and spent less time on vigilance than the adults in the two habitats, obstructive or non-obstructive. This could be mainly due to the higher feeding requirements for juveniles and their inexperience in catching and handling prey^55–57^. Alternatively, the lower state of vigilance exhibited by juveniles may also be due to their greater inexperience in detecting threats, or that they were under the ‘protection’ of their parents^57^. This latter view is supported by the fact that cranes stay within tight family groups during migration and the juveniles stay close to their parents. In addition, we also found similar declines in vigilance with increasing distance from the cranes to human disturbance in the two habitats. The minimum distance of disturbance that will trigger high degree of vigilance by cranes is 300 m, which is consistent with a similar study by Li (2011)^58^.This change reflects an adjustment of vigilance behaviour according to the degree and type of threat^58^.

### Conservation implications

Whether individual animals under tourism viewing practices chose to flee or not and the impact of that decision are crucial in evaluating the effects and sustainability of wildlife tourism^47^. For many species, there remains a lack of data on the motivations behind the decision of individual animals to tolerate the presence of humans^59^. Evidence from studies of brown bears has shown that an individual’s decision to stay or flee can be influenced by the behaviour of tourists^60^ and can even lead to displacement of individuals if wildlife tourists are permitted unrestricted close distance access to bears^61^. Due to the influence that wildlife tourists can have on the vigilance behaviour of red-crowned cranes, it is therefore important to carefully evaluate and manage all wildlife tourism practices associated with crane viewing at the YRD in order to develop guidelines for responsible crane viewing. Studies have shown that appropriately managed wildlife viewing areas (e.g. controls on the number of tourists, and scheduled viewing times) may bring direct benefits to individual animals such as improved foraging opportunities^62^. Although we did not explore the influence of varying tourist group sizes on crane behaviour, these other studies suggest that the number of wildlife tourists and the time of day tourists are permitted to view cranes should be managed carefully, particularly at important foraging sites. Wildlife tourists to the YRD should also be informed about the potential consequences of their activities to minimize any negative influence on crane vigilance behaviour, since recent studies have shown that any regulations concerning wildlife viewing practices are generally better followed when tourists understand the reasoning behind them^47^.

Our data suggests that a minimal distance of 300 m between observers and red-crowned cranes can be considered an appropriate threshold for tourism viewing practices such as photography without influencing crane vigilance in the YRD. This finding is consistent with a similar study by Li (2011)^58^. Beyond 300 m vigilance frequency, and to a lesser extent, time spent in vigilance by individual cranes remained fairly stable in both habitats. Studies of brown bears have recorded declines in vigilance behaviours in individual bears at distances > 100 m from viewing tourists^46^. Thus red-crowned cranes may continue foraging when tourist viewing sites are located > 300 m from them in these areas of the reserve and this threshold should be applied to other crane viewing sites irrespective of habitat type throughout their wintering range.

This study has another important conservational implication for the habitat of migratory red-crowned cranes. *S. salsa* vegetation is fast spreading into the *P. australis* in the Yellow River Delta^63^ and in other estuarine sites such as the Liaohe Delta in recent years. Coastal *S. salsa* vegetation is an important foraging habitat for the red-crowned crane because tidal mud-flat crabs that occupy the habitat are the predominant food resource for the wintering cranes^19^. Even though the tall *S. salsa* - *P. australis* mosaic vegetation is also used by the cranes, the high frequency of vigilance behaviours used by cranes in this habitat would have the effect of lowering overall foraging effort, and possibly reducing energy accumulation. Furthermore, crab biomass in the *S. salsa* - *P. australis* mosaic habitat is noticeably lower than in pure *S. salsa* vegetation stands (Li *et al*. unpublished data) whilst the vegetation is also exposed to a rapid invasion of *Spartina alterniflora* ^64,65^. We suspect that both the succession of the taller *P. australis* and *S. alterniflora* may not only affect the vigilance behaviour of the cranes, but can also lower the usability of these habitats because the high vegetation density that could limit the accessibility of these two habitats for the birds. We recommend that more attention should be given to the rapid loss of the *S. salsa* vegetation and that future habitat restoration efforts should provide non-obstructive and prey-rich *S. salsa* habitats for the migratory red-crowned cranes.

## Methods

### Study site and migratory crane population

The study was conducted in the coastal wetlands of the Yellow River Delta National Nature Reserve (N 37.793433°; E 119.151575°) in Shandong Province, China from 2010–2012 and 2014–2015. This reserve is now one of the most important estuarine wetland ecosystems in the eastern coastal region of China and is dominated by a highly heterogeneous landscape of different wetland habitats including natural estuarine wetlands e.g. open water, intertidal bare mudflat, *S. salsa* salt marshes, *S. salsa* - *P. australis* mosaic salt marshes; artificial wetlands (e.g. fish ponds); and restored wetlands e.g. *P. australis* freshwater marshes. This landscape mosaic provides suitable habitat for more than 90 migratory waterbird species, including 21 species each with a population that exceeds 1% of its global population^66–68^. Each year, more than half of the known continental red-crowned crane population stop over at the Yellow River Delta from October to March^19^. Previous studies have shown that these red-crowned cranes prefer the coastal *S. salsa* salt marshes and *S. salsa* - *P. australis* vegetation mosaic because of the abundant *Helice tientsinensis* tidal mudflat crab populations in these habitats, which are the primary food resource for the cranes^19^.

In this study we selected two coastal salt marshes to observe crane behaviour in response to the presence/absence of wildlife tourists. The first was an extensive area of short *S. salsa* salt marshes, averaging 10–20 cm in height, and approximately 6 km long and 1.5–3 km wide, located to the north of the yellow river. The second was an area of taller *S. salsa* - *P. australis* mosaic habitat about 5.5 km long and 2–2.5 km wide, and averaging 100–150 cm in height, located to the south of the river (see Fig. 1). The second area was previously composed of just *S. salsa* salt marsh vegetation, but has recently been colonized by *P. australis* vegetation in the high tidal zone due to locally receding tidal effects. As cranes average 160 cm in height, the *P. australis* habitat could serve as a reasonable shelter for cranes from threats when they are foraging in the *S. salsa* - *P. australis* habitat mosaic but individuals would still have to remain vigilant by scanning around with their head held upwards. In contrast, the *S. salsa* salt marshes may not serve as shelter except for when they may forage in deep tidal creeks. Both habitats are separated from inland freshwater habitats by roads that run in parallel to the coastline in the supralittoral zone of the reserve. Tides in this region are semi-diurnal with amplitude of 2–3 m, and whilst the *S. salsa* marsh is inundated semi-diurnally, the *S. salsa* - *P. australi* habitat mosaic occurs in irregularly flooded high intertidal zones and is only inundated when the tidal level is higher than the mean spring tide. During the crane’s autumn migration (late October to early December) and spring (late February to mid-March), several vehicles used by wildlife tourists appear on the roads on the edge of the crane’s feeding habitats and some of the tourists enter the wetland and approach the cranes as closely as possible in order to observe and photograph them. Further detail about the study site can be found in^19,63,66,67^.

### Behavioural observations

The basic social unit of the red-crowned crane is the family group, which typically includes two adults (a mated pair) and up to two juveniles. Juvenile cranes can be easily distinguished from adults by the brown coloured plumage on the neck and wings. Family groups are recognizable because they tend to segregate spatially throughout the wetland habitat, even when foraging, whilst family members remain closer together^55^. During the peak migration season, larger social groups sometimes form, consisting of numerous adults but with no juveniles.

Behavioural observations of red-crowned cranes were conducted from October to December and from February to March between 2010 and 2012 and between 2014 and 2015. First, we selected a focal family of cranes during daily route surveys along the roads, and their location was recorded using a GPS unit (Garmin 60 S, Garmin International, Olathe, Kansas, USA). To eliminate any observer effect on the behaviour of cranes, we selected several nearby abandoned buildings or dykes to serve as concealed observation points. On arrival at each point, we waited for several minutes before recording behavioural data in case our arrival had any influence on crane behaviour. For each family group, we randomly selected one adult and one juvenile to observe, but for some groups, we observed all family members. Behavioural observations were only conducted in good weather; when there was no rain, snow or strong wind.

Focal animal sampling was carried out using a pair of binocular (10 × 56) or a telescope (20–60 × 63). An MP3 digital recorder (TASCAM DR-100MKIII) was used to record vigilance behaviour events for 10 min until the crane flew away or it was lost in our sight from the concealed observation point. Observations continued if the cranes were obstructed by reed vegetation or when they walk into the deep creeks. Observation were recorded using a video camera (HDR-XR550E, SONY, Japan) if the distance between the cranes and the observer was less than 200 m. Vigilance behaviour was defined as a crane extending (‘stretching’) the head upwards and looking around while standing straight. We did not record other crane behaviours (e.g. foraging) since most of these behaviours were concealed by the reed patch vegetation. For each behaviour observation, we recorded the habitat type (short *S. salsa* marshes or tall *P. australis* mosaic habitat), date, time of day, age of cranes (adult or juvenile), family size, flock size and the distance from the focal crane to the nearest road (or the close approaching tourist). Flock size was defined as the number of cranes in a social flock in which distance between families was less than 50 m. We also recorded the presence or absence of wildlife tourists, which included bird watchers and nature photographers. Distance from the crane to the presence of wildlife tourists or the nearest roads in the absence of tourists was measured using the GPS locations of cranes in Google earth Pro (V7.1. 2) or by using a laser rangefinder (SCOUT DX 1000 ARC, Bushnell, USA) in the field. Most of the tourists observed wore camouflaged clothing and used off-road vehicles, and taking photographs whilst either remaining in the vehicle or on foot on the road. Only observations corresponding to the presence/absence of wildlife tourists were used in this study, and all other observations related to the absence of other non-tourist groups (e.g. fishermen) were categorized as the control group. We did not examine the effect of number of people on the vigilance behaviour of cranes because of the relatively limited sample size and tourist group numbers always ranged from 2 to5 individuals.

### Statistical analysis

Behavioural data from videos and the MP3 audio records were extracted using the EthoLog software (version 2.2)^69^. The time in which the cranes were obstructed by barriers, such as reed patches and deep tidal creeks, was calculated to represent obstruction time. Time spent on vigilant behaviour was calculated as the total time of all scanning bouts divided by the total observation time with good view of the vigilance behaviour. Frequency of vigilance (no./min) as the number of vigilance event per min of observation with no barrier obstruction. To normalize the distribution, we used the square-root transformation for vigilance time (Kolmogorov-Smirnov test: Z = 1.195, *P* = 0.115) and the vigilance frequency (Z = 0.802, *P* = 0.540), and the natural logarithm transformation for distance (Z = 1.289, *P* = 0.072). Generalized linear mixed models (GLMMs) were used to quantify the effect of the habitat and the presence of wildlife tourists on the vigilance time and the vigilance frequency, respectively, with normal distribution and an identity link function. We selected GLMMs over other modeling approaches (e.g. information theoretic model averaging) because GLMMs allow the inclusion of random effects as well as fixed effects and can simultaneously deal with non-normally distributed data^70,71^. Other confounding factors including migratory season (autumn vs. spring), observation time of the day (morning: 8:00–11:00; noon: 11:00–13:00; afternoon: 13:00–17:00), flock size (one to 50 individuals), family size (1–2 adults with 0–2 juveniles), age of the crane (adult or juvenile), the distance to human disturbance or the nearest road, and all second-order interactions with habitat and human disturbance were considered during the model building phase. Year and group ID were treated as random factors to control variation among year and the potential effects of pseudo-replication as we conducted multiple observations for individuals from the same family. All predictors were tested for collinearity (r > 0.5) using Spearman’s rank correlation index, and only the flock size and family size had potential collinearity (r = 0.496; Table S1). We build two alternative models using the flock size and family size separated, but none of them retained in the final models. The final models were constructed using the backward deletion of non-significant contributed terms. To investigate the minimum distance at which the crane would increase their vigilance efforts, we used Student-Neuman-Keuls (S-N-K) post-hoc tests with a one-way ANOVA test using five different distance categories: <200 m, 200–300 m, 300–400 m, 400–500 m and ≥500 m. All statistical analyses were performed with IBM SPSS statistical 20.0 (IBM SPSS Inc, Chicago, III). Data were presented as means ± SDs and statistical significances were considered at the *P* < 0.05 level.

### Ethical standards

This study was permitted by the Management Bureau of Yellow River Delta National Nature Reserve (issued by Yueliang Liu), and the experiments complied with the current laws of China. The experimental procedures followed the guidelines of the Animal Research Ethics Committee of the Liaoning Provincial Education Centre for Ecology and Environment, Liaoning University (No. 2013003).

## Electronic supplementary material

Supplementary Table S1
