# Supplementary material for: Habitat-dependent changes in vigilance behaviour of Red-crowned Crane influenced by wildlife tourism
Source: Sci Rep. 2017 Nov 30;7:16614. doi: 10.1038/s41598-017-16907-z (PMC5709511; doi:10.1038/s41598-017-16907-z)
Supplement: Supplementary file 1 — Supplementary Table S1 [file 41598_2017_16907_MOESM1_ESM.doc]

**Supplementary Information**

Table S1: Matrix of Spearman's rank correlation index among the eight predictor variables.

|  | Human disturbance | ln(distance) | Year | Migratory season | Time of day | Flock size | Family size | Age |
| --- | --- | --- | --- | --- | --- | --- | --- | --- |
| Habitat | -0.088 | 0.031 | 0.199 | 0.244 | 0.218 | -0.006 | -0.078 | 0.159 |
| Human disturbance |  | -0.163 | -0.246 | 0.259 | -0.011 | -0.205 | -0.038 | -0.166 |
| ln(distance) |  |  | 0.005 | -0.241 | -0.077 | -0.138 | 0.024 | -0.025 |
| Year |  |  |  | 0.393 | -0.207 | 0.160 | 0.328 | 0.070 |
| Migratory season |  |  |  |  | -0.136 | 0.386 | 0.012 | 0.037 |
| Time of day |  |  |  |  |  | 0.090 | -0.140 | <0.001 |
| Flock size |  |  |  |  |  |  | 0.496 | 0.194 |
| Family size |  |  |  |  |  |  |  | 0.218 |
